# The Long and Short of Tamoxifen Therapy: A Review of the ATLAS Trial

**Published:** 2014-01-01

**Authors:** G. Lita Smith

**Affiliations:** From University of Michigan, Ann Arbor, Michigan

## Abstract

Review of "Long term effects of continuing adjuvant tamoxifen to 10 years versus stopping at 5 years after diagnosis of oestrogen receptor-positive breast cancer: ATLAS, a randomised trial" by Davies et al. (2013), Lancet, 381, 805–816. For another perspective on the ATLAS trial, please see the article on randomization and adjusting for covariates by Dustin Dickerson starting on page 61.

Since the early 1990s, the mortality rate associated with breast cancer has been declining in all industrialized nations. This continuing decline has been attributed to the implementation of more widespread breast cancer screening practices as well as advancements in systemic therapy, including chemotherapy and adjuvant hormonal therapy (Peto, Boreham, Clarke, Davies, & Beral, 2000).

The concept of manipulating the female hormonal environment for the treatment of breast cancer dates back to the late 1800s. Several physicians at that time, including George Thomas Beatson, a Glasgow surgeon, disagreed with the prevailing theory of breast cancer as a parasitic disease. Beatson had been studying the castration of cattle for the purpose of prolonging lactation and hypothesized that manipulation of the hormonal system through oophorectomy in a premenopausal woman with advanced breast cancer might be of benefit. In an article originally published in The Lancet in 1896, Beatson reported the case studies of three young patients with advanced breast cancer who agreed to undergo bilateral oophorectomy. In his article, he described how two of the three women had remarkable decreases in their disease burden and increased palliation for some period of time following oophorectomy (Beatson, 1896).

## Hormonal Manipulation

The concept of hormonal manipulation continued to attract scientific interest. In the 1960s through the 1970s, tamoxifen, a drug that was initially evaluated (unsuccessfully) as a postcoital contraceptive medication, continued to be investigated and was found to improve progression-free survival in women with advanced breast cancer (Cole, Jones, & Todd, 1971). This led to the US Food and Drug Administration (FDA) approval of tamoxifen in the metastatic setting in 1977. In subsequent trials, tamoxifen was proven effective in the adjuvant treatment setting (Fisher et al., 1989).

Tamoxifen acts as a competitor with estrogen for the binding site of the estrogen receptor within breast tissue. Tamoxifen became the first FDA-approved selective estrogen receptor modulator (SERM) for the treatment of breast cancer. Today, it is utilized not only in the adjuvant and metastatic treatment settings, but it was also the first FDA-approved chemopreventive agent for those deemed at high risk for the development of breast cancer (Fisher et al., 1998).

## Questions of Optimal Duration

The question of optimal duration of adjuvant antiestrogen therapy, and more specifically tamoxifen, has been under ongoing investigation for decades. Many trials of varying durations of adjuvant tamoxifen were conducted. In 1998, a meta-analysis of adjuvant tamoxifen trials was reported by the Early Breast Cancer Trialists’ Collaborative Group (EBCTCG). In the 55 clinical trials reviewed (consisting of about 30,000 women), with durations of 1, 2, and about 5 years of adjuvant tamoxifen, the proportional recurrence reductions produced during about 10 years of follow-up were 21% (standard deviation [SD] 3), 29% (SD 2), and 47% (SD 3), respectively (*p* < .00001). The corresponding proportional breast cancer mortality reductions were 12% (SD 3), 17% (SD 3), and 26% (SD 4), respectively *(p* = .003; EBCTCG, 1998).

NSABP B-14 was one of the pivotal randomized, placebo-controlled clinical trials looking at 5 years of tamoxifen vs placebo in operable, estrogen receptor–positive, lymph node–negative breast cancer patients. Through 10 years of follow-up, disease-free survival (DFS) was superior in the tamoxifen arm vs. the placebo arm: 69% vs. 57% (*p* < .0001). Distant DFS was 76% vs. 67%, respectively (*p* < .0001). Overall survival trended in favor of the treatment arm: 80% vs. 76% *(p* = .02).

In 1987, an extension of B-14 commenced to investigate a longer duration of adjuvant tamoxifen. Patients on the tamoxifen arm of B-14 and disease-free at 5 years were reassigned to an additional 5 years of tamoxifen therapy vs. placebo. For patients on the placebo arm as compared with the tamoxifen arm, ongoing follow-up revealed an advantage beyond 5 years in terms of DFS, 92% vs. 86% *(p* = .003); distant DFS, 96% vs. 90% *(p* = .01); and overall survival, 96% vs. 94% *(p* = .08; Fisher et al., 1996). Therefore, the standard of care for the duration of adjuvant tamoxifen became 5 years of therapy.

## The ATLAS Trial

Despite the findings of the extension portion of the B-14 trial, additional adjuvant tamoxifen duration trials continued. The ATLAS (Adjuvant Tamoxifen: Longer Against Shorter) study was an international trial, enrolling over 12,000 patients from 1996 to 2005 from 36 countries or regions.

**Study Design** 

Eligibility criteria were having resectable disease that was completely excised, being currently on tamoxifen for 5 years, or having stopped tamoxifen within the past year and being able to easily resume therapy. Participants had to have no evidence of distant metastasis at the time of enrollment. 90% of the participants were postmenopausal women. The two treatment arms were the continuation of tamoxifen for an additional 5 years of therapy vs. placebo. No restrictions were placed on age, histology, hormone receptor status, nodal status, or other treatments (Davies et al., 2013).

**Outcomes Analysis** 

Of those study patients initially enrolled, 6,846 were confirmed to have estrogen receptor (ER)-positive disease and were included in the breast cancer–related outcomes analysis. In both study groups, 91% of survivors were still being followed 10 years after diagnosis, and 77% were still being followed 15 years after diagnosis. Comparing the tamoxifen arm with the placebo arm, the risk of recurrence in years 5 through 14 was 21.4% vs. 25.1%, respectively, and breast cancer–specific mortality was 12.2% vs. 15%, respectively, equating to an absolute breast cancer mortality reduction of 2.8%. It is important to note that longer follow-up was needed to ascertain the full benefits of extending tamoxifen treatment beyond 5 years. The reduction in the risk of breast cancer recurrence and breast cancer–related mortality was greater after year 10 following diagnosis, meeting statistical significance, and less so between years 5 and 9.

In terms of compliance to the study arms at 2 years into enrollment in the ATLAS trial, 84% of those allocated to continue on tamoxifen were compliant compared with 96% of controls (4% of control patients had initiated adjuvant hormonal therapy, predominantly tamoxifen). The researchers surmised that with 100% compliance to therapy, the benefits of longer adjuvant tamoxifen would be even greater. The EBCTCG is expected to report a meta-analysis of longer-duration tamoxifen trials, including data not only from ATLAS but from the aTTom (Adjuvant Tamoxifen Treatment Offers More) trial and other smaller trials as well.

**Side Effects** 

The most common side effects of tamoxifen therapy are postmenopausal symptoms, including hot flashes and night sweats; vaginal dryness, discharge, or irritation; and irregular menses. These toxicities can range from mild to severe, at times significantly affecting quality of life. These side effects were not discussed within the Davies et al. article, but they have been well described in previous tamoxifen trials (Day et al., 1999).

In terms of life-threatening toxicities, the two most commonly seen with tamoxifen therapy are risk of a secondary endometrial cancer and increased risk of a thromboembolic event. For the analysis of adverse events within the ATLAS trial, all 12,894 enrolled study patients were included. The most noteworthy events included the increased risk of a pulmonary embolus, for which the event rate ratio (RR) was 1.87 *(p* = .01). In terms of a secondary endometrial cancer, the event rate ratio was 1.74 *(p* = .0002). The incidence of endometrial cancer was 3.1% in the tamoxifen arm vs. 1.6% in the placebo group. Endometrial cancer–related mortality was 0.4% in the tamoxifen arm vs. 0.2% in the placebo arm. Despite the increased risk of a thromboembolic event or a secondary endometrial cancer, the expected benefits of tamoxifen therapy in terms of reduction of breast cancer recurrence and breast cancer–related mortality outweighed the expected toxicities and potential negative outcomes.

**Compliance** 

As noted earlier, the compliance rate for tamoxifen-randomized patients was around 84% at year 7 of tamoxifen therapy. This finding is not unexpected and is consistent with compliance rates noted in previously published adjuvant tamoxifen trials (Makubate, Donnan, Dewar, Thompson, & McCowan, 2013). The impact on quality of life in terms of toxicities such as postmenopausal symptoms is not to be underestimated. Concern about life-threatening adverse events such as thromboembolic events or secondary endometrial cancers has also kept patients from initiating and/or completing adjuvant tamoxifen therapy. If the absolute benefit in terms of decrease in breast cancer–related mortality of an additional 5 years of tamoxifen therapy is only 2.8%, a balanced discussion needs to occur weighing the benefits of therapy vs. the impact on quality of life and potential life-threatening toxicities.

## Other Antiestrogen Therapies

Other antiestrogen therapy options, such as aromatase inhibitors (AIs), and results from clinical trials in which patients switch from tamoxifen to an AI or vice versa with varying schedules and durations of therapy have confounded a clear path as to the optimal drug(s) and duration of adjuvant antiestrogen therapy.

One pivotal trial in this arena was the ATAC (Arimidex, Tamoxifen, Alone or in Combination) trial, which provided data in favor of an AI vs. tamoxifen taken for 5 years (Cuzick et al., 2010). The AI anastrozole was found to be superior to tamoxifen in terms of improving DFS in postmenopausal women (hazard ratio [HR], 0.86; 95% confidence interval = 0.78–0.95;* p* = .003).

MA.17 was a clinical trial that randomized postmenopausal women to 5 years of letrozole therapy vs. placebo following 5 years of tamoxifen therapy (Goss et al., 2005). With a median follow-up of 30 months, women on letrozole saw improvement in terms of DFS compared to placebo (HR, 0.58; *p* < .001). There is now an extension portion of MA.17 randomizing those on the letrozole arm of MA.17 to an additional 5 years of therapy vs. placebo. The results of the extension portion of MA.17 are not yet known.

In 2010, the American Society of Clinical Oncology (ASCO) updated its adjuvant breast cancer guideline to recommend that all postmenopausal women with hormone receptor–positive breast cancer use an AI either alone or before or after tamoxifen to reduce their risk of recurrence. The guideline updates went on to say that women may also use AIs for extended periods, after 5 years of tamoxifen therapy, to lower their risk of recurrence (Burstein et al., 2010).

## Conclusion

For postmenopausal women, the decision to initiate tamoxifen or AI therapy up front may be based on stage of disease, risk of recurrence, age, comorbidities, or personal choice. If tamoxifen is initially started, the ASCO guidelines recommend switching to an AI at some time point during the course of antiestrogen therapy. For premenopausal women, tamoxifen, now in a duration of 10 years, may provide a viable option to further decrease the risk of breast cancer recurrence. The expectation is that more clinical trials and longer follow-up will continue to guide clinicians regarding the optimal therapy and duration of adjuvant antiestrogen therapy.
